# The relationship between glycemic control, beta2-microglobulin and inflammation in patients on maintenance dialysis treatment

**DOI:** 10.1186/s40200-015-0162-1

**Published:** 2015-04-23

**Authors:** Vaia D Raikou, Despina Kyriaki

**Affiliations:** 1st Department of Medicine - Propaedaetic, National and Kapodistrian University of Athens, School of Medicine, 17 Agiou Thoma, Αthens, Greece; Department of Nuclear Medicine, General Hospital “LAΪKO”, Αthens, Greece

**Keywords:** Glycemic control, Beta2-microglobulin, Inflammation, Dialysis

## Abstract

**Background:**

Hyperglycemia appears to play a significant role on the inflammatory cytokines production. Beta2-microglobulin (beta2M) is accumulated in the circulation of dialysis patients. We studied the relationship between glycemic control defined by glucose serum concentrations and insulin resistance, beta2M and markers of inflammation in patients on renal replacement therapies with or/and without diabetes mellitus.

**Methods:**

We enrolled 96 dialyzed patients, 62 males and 34 females. The treatment modalities which were applied were : regular hemodialysis (HD, n = 34), predilution hemodiafiltration (HDF, n = 42) and peritoneal dialysis (PD, n = 20). Dialysis adequacy was defined by Kt/V for urea.Beta2M and insulin serum concentrations were measured by radioimmunoassays. hsCRP and TNF-α serum concentrations were measured by ELISA. Insulin resistance was calculated using the homeostasis model assessment of insulin resistance (HOMA-IR).We examined the association of elevated serum glucose with inflammatory factors and we built a multivariable model to investigate if glucose could be a potential determinant of beta2M serum levels.

**Results:**

Serum glucose was positively correlated with beta2M and TNF-α (r = 0.320, p = 0.002 and r = 0.215, p = 0.03 respectively).We observed significant association between the patients with higher serum glucose concentrations and the patients with greater beta2Μ concentrations (x^2^ = 4.44, p = 0.03). Multivariable model showed that glucose acts as a significant independent determinant of beta2M adjusting for age, gender, dialysis modality and metabolic acidosis status.

**Conclusions:**

The elevated glucose concentrations were positively associated with both, greater beta2M serum concentrations and up-regulated inflammatory procedure in dialysis patients with or/and without diabetes mellitus.

## Introduction

Beta2-microglobulin (beta2M), which is one of the major histocompatibility complex class I molecules, is accumulated in the circulation of dialysis patients [1].

It has been shown that hyperglycemia play a significant role on the development of microangiopathy, endothelial dysfunction and on the inflammatory cytokines production related to atherosclerosis [2]. Poor glycemic control is associated with the development of comorbidities such as coronary artery disease and myocardial infarction causing sudden cardiac death in the general population [3]. Also, glycemia influences the electrolyte balance, the function of potassium and calcium channels, and sympathetic activity resulting in arrhythmogenesis and sudden death particularly of patients in end stage of renal disease (ESRD) [4].

However, it is not clear whether patients in the end stage of renal disease (ESRD) with or/and without diabetes mellitus benefit from a strict glycemic control [5]. Few studies have been reported on the effect of intensive glucose control in the later stages of chronic kidney disease [6]. Despite, it has been reported that intensive glycemic control reduced the risk of microangiopathy in both type 1 and 2 diabetes [7], previous trials evaluating the effect of glucose control have provided conflicting results on the rate of decrease in glomerular filtration rate (GFR) or ESRD in diabetics. The Kidney Disease Outcomes Quality Initiative guidelines suggested that lowering the glycated haemoglobin (HbA1c) level to <7.0% might reduce the rate of decrease in renal function [8].On the other hand, in previous follow-up study of the Action to Control Cardiovascular Risk in Diabetes Study Group (ACCORD) trial, the outcome of ESRD was not significantly different between the intensive and standard glucose control groups [9].

In this study we focused on the relationship between glycemic control defined by glucose serum concentrations and insulin resistance, beta2M and markers of inflammation in patients undergoing maintenance dialysis with or/and without diabetes mellitus.

## Methods

### Patients

We studied 96 dialyzed patients at Nephrology Department of “Laiko, University General Hospital of Athens” and Renal Unit of “Diagnostic and Therapeutic Center of Athens Hygeia SA”.62 males and 34 females participated in this study, on mean age 62.1 ± 14.27 years old. Patients with active infection, malignancy, glucocorticoid therapy or lymphoproliferative disease were excluded from our study.

The treatment modalities which were applied were : regular hemodialysis (HD, n = 34), on-line- predilution hemodiafiltration (on-l HDF, n = 42) and peritoneal dialysis (PD, n = 20).The median time on hemodialysis was 5 years, interquartile range 3–10 years and the mean time on peritoneal dialysis was 2.8 ± 1.61 years.

The hemodialysis treatment was performed 3-times weekly with a dialysis time of 3.5-4 h per session, a filter of 1.5-2 m^2^ surface area and a blood flow of 350–400 ml/min.A bicarbonate-based ultrapure buffer dialysis solution was used with a dialysate flow rate of 500-600 ml/min, a calcium concentration of 1.50-1.75mmol/L, a sodium concentration of 138-145mmol/L and low molecular weight heparin as anticoagulant therapy.

We used exclusively high-flux synthetic dialysis membrane, defined by a ultrafiltration coefficient >20 ml/h [10].Dialysis dose was defined by Kt/V for urea, which was calculated according to the formula of Daugirdas [11]. Patients were excluded if they had Kt/V for urea <1.2.

All patients on peritoneal dialysis were following continuous ambulatory peritoneal dialysis (CAPD) with 4 changes per day using a combination of 2 changes of 2 L of hypertonic glucose-based solution (3.86% glucose; Baxter Healthcare) and 2 changes of 2 L of semi-hypertonic glucose solution (2.5% glucose; Ariti; Bieffe Medital S.p.A.). Dialysis dose was defined by Kt/V/week for urea in this group of patients and the patients, who had more than 2 – 3 events of peritonitis per year, were excluded from our study.

20 hemodialyzed patients and 15 peritoneal dialyzed patients excreted up to 100 ml of urine per day.None of the enrolled patients was receiving hypolipidemic medicines.Only calcium-free phosphate binders were prescribed. The total of our subjects was on erythropoetin-a or-β therapy.

The underlying renal disease was hypertensive nephrosclerosis (n = 31), chronic glomerulonephritis (n = 28), polycystic kidney disease (n = 12), diabetic nephropathy (n = 11), and other/unknown (n = 14).

### Approval and consent

The study was approved by the ethics committee of the Hospitals “Laiko, University General Hospital of Athens” and Renal Unit of “Diagnostic and Therapeutic Center of Athens Hygeia SA”.Written informed consent was obtained from all subjects.

### Blood collection

Blood samples were obtained by venipuncture in the peritoneal dialyzed patients in a twelve hours fasting state.In hemodialyzed patients blood was drawn just before the start of the mean weekly dialysis session also in a twelve hours fasting state from the vascular access.In the end of the treatment the blood pump speed was reduced to <80 ml/min and blood samples was obtained at 2 min post-dialysis from the arterial dialysis tubing for the calculation of the adequacy of dialysis by kt/V for urea. The blood samples were centrifuged, and kept at a temperature of −80° C.

In each subject, three sequences of samples were received for the serum glucose measurements, and their average was used for statistical analysis.

### Laboratory measurments

Albumin and serum glucose concentrations were measured by biochemical analyzer (Architect, ci 16200, Abbott).Hematocrit, hemoglobin and monocytes blood cells values were measured by hematological analyzer (Sysmex, xt-4000i, Roche).

The concentrations of beta2-microglobulin and insulin were measured by radioimmunoassays (Immunotech by Beckman, Czech Republic and BioSource Europe SA, Belgium respectively).

Insulin resistance was calculated using the homeostasis model assessment of insulin resistance (HOMA-IR) [12].

High sensitivity C-reactive protein (hsCRP) and serum tumor necrosis factor-a (TNF-a) concentrations were measured using enzyme linked immunoabsorbed assays (ΕLISA, Immundiagnostik AG., Germany and Ani Biotech Oy, Orgenium, Finland respectively) according to manufacturer’s specifications.

Normalized protein catabolic rate for dry body mass (nPCR) was calculated from the urea generation rate [13].Body mass index (BMI) was obtained from height and post-dialysis body weight.

Metabolic acidosis was defined by serum bicarbonate levels, which were measured using a blood gas analyzer (Roche, cobas b 121 system) taking care of the blood specimens [14].

### Data analysis

Data was analyzed using SPSS 15.0 statistical package for Windows (SPSS Inc, Chicago, Illinois) and expressed as mean ± standard deviation or as median value ± interquartile range for data that showed skewed distributions; differences between mean values were assessed by using paired-t test and Mann–Whitney U test. Correlations between variables were defined by Pearson and Spearman coefficient and p values less than .05 were considered significant. x^2^ analysis was used for the correlation between categorical variables.We built a linear regression analysis to investigate if glucose serum concentrations could be a potential determinant of beta2M serum levels in dialysis patients adjusting for age, gender, dialysis modality and metabolic acidosis state.

## Results

Characteristics and demographical characteristics of the studied population at the time of inclusion are listed in Tables 1 and 2.

**Table 1:** **Characteristics of the studied population, n = 96 [Haemodialysis, HD, n = 76, Peritoneal dialysis, PD, n = 20 (62 males/34 females)]**

***Characteristic***	***Minimum***	***Maximum***	***Mean/median***	***SD/*** **interquart range**		
Age (years)	24	87	62.10/	14.27/		
Haemodialysis duration (years)	0.5	27	/5.0	/3 - 10		
Peritoneal dial duration (years)	1	6	2.8/	1.61/		
Body mass index (Kg/m^2^)	18.10	43.50	25.08/	3.86/		
Kt/V/day for HD patients (n = 76)	1.2	2.01	/1.29	/1.25 – 1.49		
Kt/V/week for PD patients (n = 20)	1.7	2.58	2.14/	0.27/		
Normalized protein catabolic rate (nPCR, g/Kg/day) (n = 96)	0.97	3.41	2.23/	0.616/		
Urine volume (ml/day)	100	1500	337.35/	305.8/		
Serum bicarbonate levels (mmol/L )	14.8	25.8	20.64/	2.59/		
beta2-microglobulin (mg/L)	8.29	138.0	/25.98	/16.1 – 32.7		
hsCRP (mg/L)	0.12	21.35	8.65/	5.9/		
insulin (μU/ml)	3.16	110	/15.6	/11.3 – 29.8		
ΗΟΜΑ-ΙR (mmol/L)	1.00	50.88	/3.6	/2.1 – 7.1		
TNF-α (pg/ml)	0.10	170.4	/0.18	/0.0 – 31.0		
Glucose (mg/dl)	60	201	97.7/	26.6/		
Albumin (gr/dl)	1.4	4.6	3.88/	0.44/		
Htc (%)	23.9	45.3	35.5/	4.1/		
Hemoglobin	7.97	15.1	11.8/	1.37/		
Monocytes (K/μl )	0.10	1.20	0.53/	0.23/	1.08	.43

**Table 2:** **Demographical characteristics of studied patients, n = 96, (62 males/34 females)**

***Characteristic***	***Valid percent***
BMI > 25	43.4
Hypertension (yes)	32.3
Diabetes mellitus (yes)	11.5
Smoking (yes)	21.9
Left ventricular hypertrophy (yes)	56.3
Coronary disease (yes)	31.3

We divided the studied patients in two groups according to serum glucose concentrations ( greater, n = 36 or lower, n = 60 than 100mg/dl ).Comparing these groups between them, we observed that the patients with higher glucose concentrations (n = 36) had significantly increased beta2M serum concentrations, insulin levels, HOMA-IR and number of circulating blood monocytes than the patients with lower serum glucose values (p < 0.05).BMI, hsCRP and TNF-α values were also greater in patients with higher glucose concentrations (Table 3).

**Table 3:** **Differences between groups of dialysis patients according to glucose serum concentrations**

	***Patients with glucose serum concentrations less than 100mg/dl (n = 60)***	***Patients with glucose serum concentrations more than 100mg/dl (n = 36)***
beta2-microglobulin	28.3	40.4*
insulin	16.5	29.8*
ΗΟΜΑ-ΙR	3.39	9.98*
Monocytes	0.49	0.60*
hsCRP	8.24	9.33
TNF-α	20.8	24.05
BMI	24.7	25.6

*p < 0.05.

**Figure 1:**
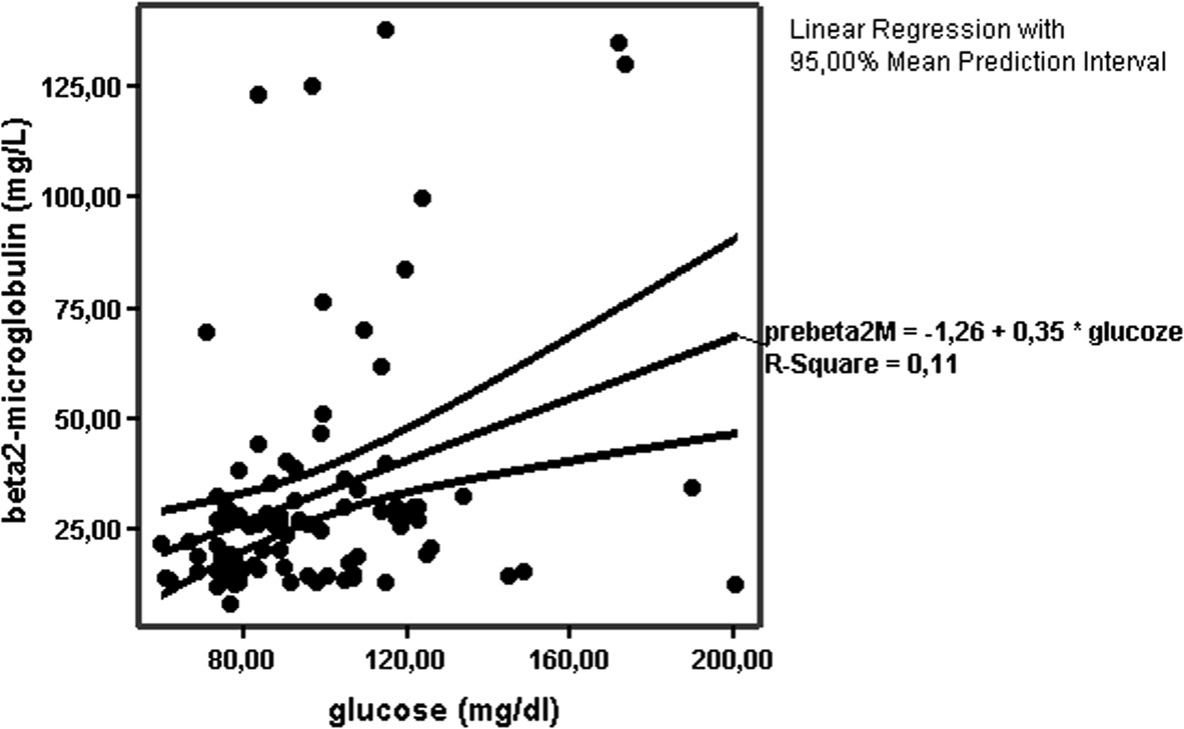
Positive correlation between glucose and beta2-microglobulin serum concentrations (r = 0.320, p = 0.002).

In total patients serum glucose was positively correlated with beta2M (Figure 1), insulin, HOMA-IR, TNF-α and monocytes (r = 0.320, p = 0.002, r = 0.558, p = 0.001, r = 0.736, p = 0.001, r = 0.215, p = 0.03 and r = 0.302, p = 0.005 respectively).Beta2M was also positively associated with hsCRP (r = 0.257, p = 0.01) and monocytes (r = 0.438, p = 0.001).

**Figure 2:**
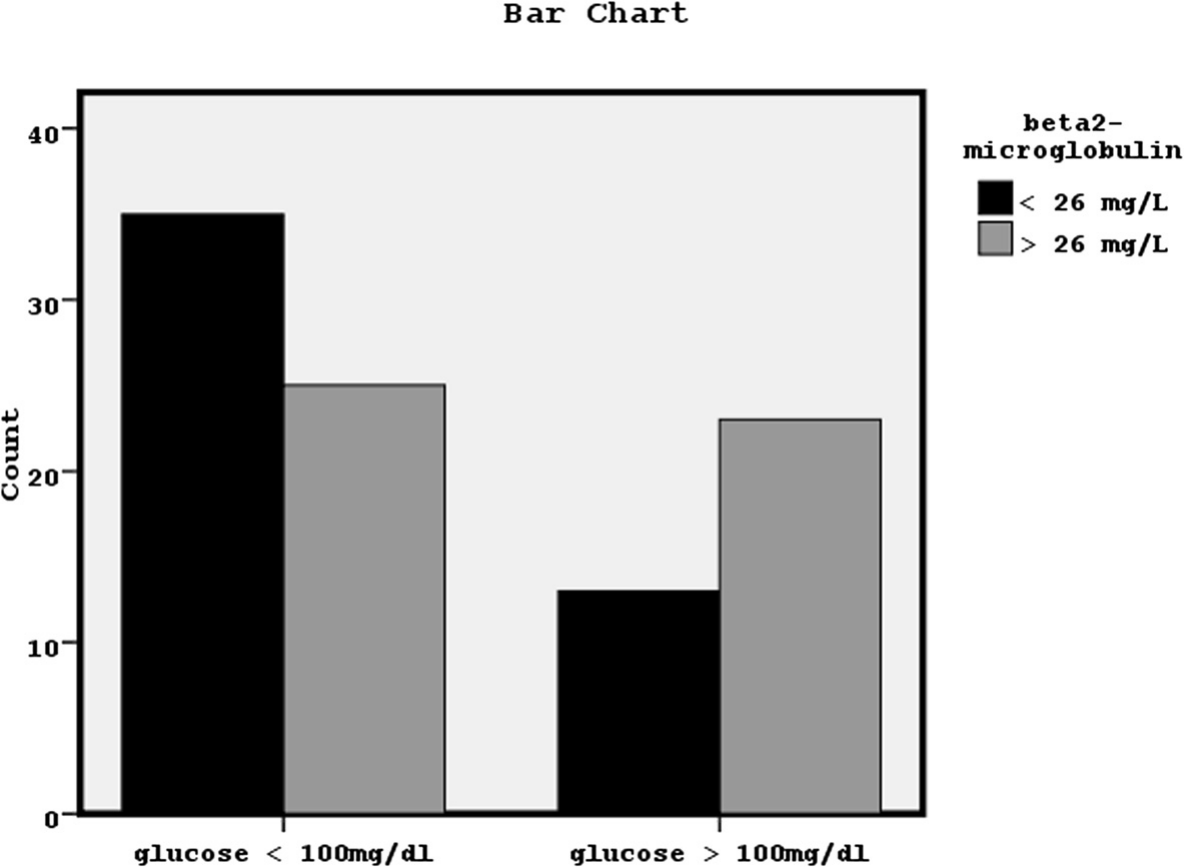
Significant association between the patients with higher serum glucose concentrations (more than 100mg/dl) and the patients with greater serum beta2-microglobulin concentrations (more than the median value of 26 mg/L) (x^2^ = 4.44, p = 0.03).

Additionally, x^2^ analysis showed significant association between the patients with higher serum glucose concentrations and the patients with greater serum beta2Μ concentrations, using the median value of beta2M equal to 26mg/L, as dividing value (x^2^ = 4.44, p = 0.03) (Figure 2).

Linear regression analysis showed that glucose acts as a significant independent determinant of beta2M serum levels after adjustment for age, gender, dialysis modality and metabolic acidosis state (Table 4).

**Table 4:** **Glucose as a potential independent determinant of beta2-microglobulin serum concentrations in studied dialyzed patients**

	**Beta**	**t**	**Sig.**	**Lower**	**Upper**
age	−0,032	−0,258	0,797	−0,377	0,291
gender	−0,1	−0,852	0,397	−13,318	5,355
Dialysis modality	−0,017	−0,133	0,894	−11,524	10,082
Serum bicarbonate	−0,024	−0,184	0,854	−2,088	1,736
glucose	0,361	3,04	0,003*	0,09	0,434

*: p < 0.05.

## Discussion

In present study, we observed a positive correlation between serum glucose consentrations and inflammatory factors, as beta2M, TNF-α and circulating monocytes.Additionally, the patients with higher serum glucose had significantly higher beta2M serum concentrations, insulin and insulin resistance values, as well as significantly more circulating monocytes, than the patients with lower serum glucose concentrations. hsCRP and TNF-α levels were also greater in higher glucose group of patients.

It has been already reported that hyperglycemia up-regulates inflammatory procedure by the excess generation of inflammatory cytokines and reactive free radicals, causing oxidative stress [2]. Also, hyperglycemia induces the production of the advanced glycation end products (AGEs), which exert oxidative stress, or can be a consequence of oxidative stress [15].These toxins are profibrotic and directly involved in the pathogenesis of the inflammatory response syndrome and in vascular complications. In the meantime, the generation of elevated AGEs during hyperglycemia modifies beta2M.

Beta2M , which has been increasingly investigated as a diagnostic marker of kidney function, is accumulated in the circulation of dialysis patients and its role on immunity and inflammation has been already reported [16].In agreement, in present study we observed significantly positive association between beta2M, circulating monocytes and hsCRP. Also, AGE-modified beta2M (AGE-beta2M) related to hyperglycemia may accelerate the inflammatory procedure and may directly participate in the pathobiology of amyloid formation in vessels wall by stimulating chemotaxis of monocytes and synthesis of cytokines from macrophages (interleukin-1β, TNF-α, and interleukin-6) [17,18].

Another pathophysiological mechanism, which exerts the oxidative stress and the increased production of AGEs during hyperglycemia in this population of patients, is metabolic acidosis, which is a common condition particularly in end-stage renal disease patients [19]. Metabolic acidosis promotes inflammation releasing cytokines and it is connected to many adverse effects of these patients including glucose metabolism disorder, increased insulin resistance and increased accumulation of beta2Μ [20]. Maintenance dialysis therapies are often unable to completely correct the base deficit.

Additionally, in this study using a multivariable model we noted that glucose serum concentrations can be a strong independent determinant of beta2M serum concentrations adjusting for age, gender, dialysis modality related to dialysis adequacy and acidosis status.

Controversially to the findings of present study, previous study found no association between beta2M and markers of diabetes mellitus, as glucose or glycated hemoglobin (HbA1c), using a multivariate regression model adjusting for age, gender and kidney function (eGFR) in 1302 elderly healthy individuals, despite the relationship of beta2M with CRP, systolic blood pressure, total cholesterol and current smoking was found significant [21].In dialysis patients special conditions permanently hold, which are associated to up-regulation of inflammatory microenvironment, as acidosis state and oxidative stress, uremic toxicity, increased insulin resistance and hypertension due to fluid overload [22,23]. These special conditions influence and are influenced by hyperglycemia worsening its complications, thus hyperglycemia may be more hurtful in dialysis patients comparatively to healthy subjects, even though the studied healthy subjects are on an old age.

On the other hand, the renal function in dialysis patients is exclusively represented by dialysis adequacy defined by Kt/V for urea, related to following dialysis modality, used dialysis membrane and duration of hemodialysis session, in addition to the residual kidney function, when it exists.Dialysis adequacy mainly regulates beta2M serum concentrations in these patients, in combination to the contribution of existing residual kidney function [24], thus beta2M reflects the obtained dialysis adequacy and serves as a surrogate marker of other middle-molecules uremic toxins [25].Because of the obtained dialysis adequacy is difficult to be equal to normal renal function, beta2M values are commonly higher in dialysis patients than in healthy subjects.

It could be suggested that a hurtful hyperglycemia, as hyperglycemia in uremic and oxidative environment, can determine the already elevated levels of beta2M, which, in the meantime, is modified during hyperglycemia becoming more toxic and accelerating the inflammatory procedure.This could be the explanation for the positive association between glucose and beta2M explored in present study, in opposite to the above reported previous study, that studied elderly healthy subjects, whose the conditions are absolutely different comparatively to dialysis patients.

Based on the findings of this study, we could support that the elevated beta2M serum concentrations predispose to a up-regulation of the inflammatory procedure, which is associated with the increased glucose serum concentrations in dialysis patients.On the other hand, the elevated glucose serum concentrations were found as a strong determinant of beta2M serum levels in dialysis patients, thus a better glycemic control in combination to a better beta2M clearance obtained by a good dialysis adequacy should be very beneficial for this population of patients, resulting in down-regulation of inflammation.

## Conclusions

The elevated glucose concentrations were positively associated with both, greater beta2M serum concentrations and up-regulated inflammatory procedure in dialysis patients with or/and without diabetes mellitus.
